# Facilitating gene editing in potato: a Single-Nucleotide Polymorphism (SNP) map of the *Solanum tuberosum* L. cv. Desiree genome

**DOI:** 10.1038/s41598-020-58985-6

**Published:** 2020-02-06

**Authors:** François Sevestre, Maud Facon, Fabrice Wattebled, Nicolas Szydlowski

**Affiliations:** 1 Univ. Lille, CNRS, UMR8576 – UGSF – Unité de Glycobiologie Structurale et Fonctionnelle, F-59000 Lille, France; 2 Univ. Lille, CNRS, USR 3290 – MSAP – Miniaturisation pour la Synthèse l’Analyse et la Protéomique, F-59000 Lille, France

**Keywords:** Molecular engineering in plants, Transgenic plants

## Abstract

Genome editing is a powerful tool for plant functional genomics allowing for multiallelic targeted mutagenesis. The recent development of Clustered Regularly Interspaced Short Palindromic Repeats (CRISPR)/CRISPR associated protein 9 (Cas9) systems for gene editing in plants allows for simple, cost-effective introduction of site-specific double-stranded DNA breaks. The nuclear genomes of a homozygous doubled-monoploid potato clone (DM) and a heterozygous diploid clone (RH) have been sequenced in 2011. However, common potato cultivars display a highly heterozygous autotetraploid genome thus complicating target design for tetra-allelic gene editing. Here, we report on the SNP physical map of the widely used *Solanum tuberosum* L. cv. Desiree and on the position of the diverse indels providing an essential tool for target design in genome editing approaches. We used this tool for designing a specific gRNA and successfully knocking-out a newly discovered starch synthase gene (*SS6*) in potato. Resequencing data are publicly available at the Sequence Read Archive of the NCBI (accession number: PRJNA507597) and will represent a valuable resource for functional genomic studies of various metabolic pathways, cell and plant physiology as well as high-throughput reverse genetics in potato.

## Introduction

Potato belongs to the Solanaceae family such as tomato, eggplant, pepper or tobacco. About 200 tuber-bearing potato species are classified within the subgenus *Potatoe* of the genus *Solanum*. Common potato corresponds to the subspecies *S*. *tuberosum* ssp. *tuberosum* that is cultivated for its starch-, vitamin-rich tubers. Potato is the fourth most important staple crop in the world with 377 million tonnes produced in 2016 thus substantially contributing to global food safety (www.fao.org). Similar to other staple crops comprising rice, wheat or maize, important research effort is put into improving potato in a climate changing context concomitant with increasing global population. This includes biofortification and yield increase, pest protection, abiotic stress adaptation as well as post-harvest conservation and processing. As an example, starch metabolism remains intensively investigated since it influences the rheological properties of processed food or non-food potato-based products^1^. In a recent study, we identified a series of unknown proteins in association with potato starch granules by shotgun mass spectrometry^2^. Among these proteins, a yet unknown isoform of starch synthase, SS6, showed very high potential to be a key enzyme of the starch biosynthetic pathway. Common potato species are autotetraploid (2*n* = 4*x* = 48) thus complicating traditional breeding. Traits of interest follow tetrasomic inheritance patterns and chromatid segregation may occur during crossing. Substantial work was performed to produce diploid clones and facilitate genetic research. In particular, this led to the genome release of a homozygous doubled-monoploid (DM) and a heterozygous diploid (RH) clone^3^. This important milestone for genetic studies and potato improvement revealed that inbreeding depression is likely related to the high genome plasticity of the species and the presence of numerous potentially deleterious mutations^3^.

A series of transgenic strategies were employed both in functional genomic studies and crop breeding comprising insertional mutagenesis with the use of transposons or Agrobacterium mediated T-DNA insertions^4^. In both cases, multiple insertions can occur within plant genomes thus leading to undesired secondary mutations. Moreover, complete gene knockout (KO) in polyploids requires plant sexual propagation for at least two generations involving potential loss of valuable fixed traits. In the highly heterozygous tetraploid potato, such approach is laborious and time consuming. On the other hand, sequence-specific DNA nucleases were used to introduce double strand breaks (DSBs) that are repaired by non-homologous end joining (NHEJ) or homologous recombination (HR)^5^. Zinc finger nucleases (ZFNs) and transcription activator-like effector nucleases (TALENs) are chimeric proteins composed of a sequence-specific domain fused to a non-specific nuclease (Fok1) that have proven successful in editing the genome of different plant species comprising potato^6–9^. Application of the recently developed Clustered Regularly Interspaced Short Palindromic Repeats (CRISPR)/CRISPR associated protein 9 (Cas9) technology increased rapidly over the past years^10^. Contrary to TALENs and ZFNs, CRISPR/Cas9 do not require specific protein engineering and was efficient for introducing deletions, insertions and performing multiplex genome modifications of plants^11^. The CRISPR/Cas9 system involves a single monomeric nuclease and a chimeric RNA composed of a 20-bp segment conferring site specificity as well as a scaffold sequence necessary for Cas binding^11^. Proof of concept for CRISPR mutagenesis in the tetraploid potato was achieved with the use of a *U6* promoter upstream of the gRNA^12,13^. In order to avoid integration of foreign DNA in the genome, strategies using transient expression of the Cas9 and the gRNA or transfection of CRISPR-Cas9 synthetic ribonucleoproteins into potato protoplasts were implemented^14,15^. Recently, the granule-bound starch synthase (GBSS) was inactivated by CRISPR-Cas9 base editing of the conserved catalytic KTGGL-encoding locus in the tetraploid potato cultivar Desiree^1^.

Regardless of the genome editing system, inducing homozygous DSBs in tetraploid potato lines requires targeting of a SNP-/insertion-/deletion–free DNA region. Haplotype diversity in potato was investigated by sequence comparison of the RH and DM genomes^3^. The latter revealed that SNPs occur every 40 bp and that indels, of an average length of 12.8 bp, arise every 394 bp. This frequency was even higher when comparing both RH haplotypes with 1 SNP per 29 bp and 1 indel per 253 bp. Furthermore, SNP-based analysis of 23 North American potato modern cultivars showed a population diversity (π) of 0.0105, which was significantly higher than previous resequencing studies of crops^16^. Therefore, determining both allelic variation of the target region (polymorphisms intrinsic to the studied potato line) and sequence divergence from the DM reference genome is essential for designing targets leading to homozygous gene editing in common tetraploid potato lines.

Here we report on the NGS-based resequencing analysis of the widely used *Solanum tuberosum* L. cv. Desiree. About 100 million reads were aligned on the DM reference genome prior to SNP-/Indel-calling. Combining variations within the genome of the potato cultivar Desiree (Desiree vs. Desiree) and variations between the DM reference genome and that of cv. Desiree (Desiree vs. DM), our analysis showed occurrences of 1 SNP/68 bp, 1 insertion/1173 bp and 1 deletion/1753 bp. Furthermore, we provide with a physical map of polymorphisms occurring within the assembled regions of each of the 12 Desiree chromosomes illustrating the heterogeneity of polymorphisms. In particular, polymorphism enrichment could be observed at the edges of chromosomes 7 and 10. On the other hand, variations were poorer in the central region of chromosome 7 and 12 as well as in the regions ranging from 10 to 60 Mb and 15 to 30 Mb of chromosome 1 and 3, respectively. Increasing the number of potential targets is essential to improve the success rate in tetra-allelic mutagenesis or in specific approaches such as CRISPR-driven base editing. Thus, polymorphisms intrinsic to the Desiree genome (Desiree vs. Desiree) were also investigated by filtering out divergences from the DM reference genome (Desiree vs. DM). This analysis revealed that 70% and 67% of the SNPs and indels, respectively, occurred within the genome of Desiree. Most importantly, this work represents an essential tool for high throughput functional genomic studies of the potato. This was illustrated by the knock-out mutagenesis of *SS6*, a recently discovered starch synthase isoform most likely involved in starch biosynthesis^2^.

## Results

In order to locate SNPs, insertions and deletions occurring in a commonly used tetraploid potato cultivar, leaf genomic DNA was isolated from *in vitro* cultivated cv. Desiree plants prior to genome resequencing. 99,713,534 reads of 2 × 150 bp were generated and mapped on the *S*. *tuberosum* Group Phureja DM1-3 Version 4.03 Pseudomolecule genome Sequence (http://solanaceae.plantbiology.msu.edu/pgsc_download.shtml)^3^. The percentage of mapped reads was 83.9%, corresponding to a mean coverage of almost 30X. This sequencing depth was similar to previous resequencing studies and allowed for confident identification of polymorphisms in tetraploid potato plants^3,16^. Variant calling analysis identified 11,939,720 SNPs, 707,507 insertions and 470,182 deletions. These variations included local sequence divergence between cv. Desiree and the DM reference (Desiree vs DM) and sequence polymorphism occurring between haplotypes of the Desiree tetraploid genome (Desiree vs. Desiree). The occurrence and distribution of these variations as well as the different types of nucleotide substitutions are summarized in Table 1. These data show that polymorphisms occur every 59 bp on average with SNPs being the predominant type of variation (Table 1). At the whole genome level, an average occurrence of one SNP every 68 bp was observed while indels were less frequent with one insertion or deletion per 1173bp and 1753bp, respectively (Table 1). Furthermore, SNP occurrence was most prevalent on chromosome 11 with one SNP every 55bp while only one SNP per 92bp was observed on chromosome 10 (Table 1).

**Table 1:** Genetic variations in the genome of *Solanum tuberosum* cv. Desiree mapped to that of Solanum Phureja (DM).

	Chromosome length (Mbp)	Deletions	Insertions	SNPs	Occurrence (1 variation in every × bp)			Transition		Transversion			
					Deletion	Insertion	SNP	A/G	C/T	A/C	T/A	C/G	G/T
Ch00	48,012,048	13,194	19449	426908	3639	2469	112	124,265 (29.9%)	125,239 (30.1%)	44,168 (10.6%)	54,178 (13.0%)	24,484 (5.9%)	43,681 (10.5%)
Ch01	88,663,952	57,427	88,370	1,541,312	1544	1003	58	458,504 (30.6%)	459,233 (30.7%)	145,797 (9.7%)	207,069 (13.8%)	80,531 (5.4%)	145,258 (9.7%)
Ch02	48,614,681	34,589	52,865	756,027	1405	920	64	223,952 (30.6%)	223,808 (30.6%)	68,087 (9.3%)	108,617 (14.9%)	38,255 (5.2%)	68,489 (9.4%)
Ch03	62,290,286	37,547	59,237	811,542	1659	1052	77	241,589 (30.8%)	240,680 (30.7%)	72,951 (9.3%)	116,763 (14.9%)	40,263 (5.1%)	72,989 (9.3%)
Ch04	72,208,621	44,063	61,917	1,089,325	1639	1166	66	325,072 (30.8%)	323,821 (30.7%)	103,310 (9.8%)	141,941 (13.4%)	57,996 (5.5%)	103,636 (9.8%)
Ch05	52,070,158	34,988	50,794	920,998	1488	1025	57	277,313 (31.1%)	275,880 (31.0%)	85,192 (9.6%)	119,421 (13.4%)	46,925 (5.3%)	85,859 (9.6%)
Ch06	59,532,096	42,176	65,729	1,008,413	1412	906	59	303,836 (31.0%)	302,651 (30.8%)	92,649 (9.4%)	138,366 (14.1%)	51,404 (5.2%)	92,783 (9.5%)
Ch07	56,760,843	35,827	53,346	880,487	1584	1064	64	262,797 (30.7%)	263,627 (30.8%)	83,058 (9.7%)	118,261 (13.8%)	45,261 (5.3%)	82,357 (9.6%)
Ch08	56,938,457	36,378	55,110	961,271	1565	1033	59	288,083 (30.9%)	289,812 (31.1%)	89,489 (9.6%)	127,602 (13.7%)	48,198 (5.2%)	89,661 (9.6%)
Ch09	61,540,751	37,856	55,536	1,020,000	1626	1108	60	304,203 (30.7%)	303,888 (30.6%)	98,053 (9.9%)	133,370 (13.4%)	53,481 (5.4%)	98,773 (10.0%)
Ch10	59,756,223	28,785	40,510	649,220	2076	1475	92	195,481 (31.1%)	194,222 (30.9%)	59,943 (9.5%)	85,473 (13.6%)	33,620 (5.3%)	59,798 (9.5%)
Ch11	45,475,667	31,574	49,776	822,217	1440	914	55	248,611 (31.2%)	247,285 (31.0%)	74,917 (9.4%)	111,633 (14.0%)	40,400 (5.1%)	75,111 (9.4%)
Ch12	61,165,649	35,778	54,868	1,052,000	1710	1115	58	315,281 (30.8%)	315,028 (30.8%)	100,479 (9.8%)	135,438 (13.3%)	55,857 (5.5%)	100,083 (9.8%)
Total	773,029,432	470,182	707,507	11,939,720	1753	1173	68	3,568,987 (30.8%)	3,565,174 (30.8%)	1,118,093 (9.7%)	1,598,132 (13.8%)	616,675 (5.3%)	1,118,478 (9.7%)

The variants correspond to both local sequence divergence between cv. Desiree and the DM reference as well as to sequence polymorphism occurring between haplotypes of the Desiree tetraploid genome.

The genome-wide mean transition (A/G or T/C) to transversion (A/C, T/A, C/G or G/T) ratio was 1.603, which was consistent with a previous work^17^. Both types of transition (30.8% of the total SNPs) appeared at a similar frequency, with 3,568,987 A/G and 3,565,174 C/T transitions at the genome scale. Conversely, transversion occurrence varied according the nature of the substitution. T/A was the most frequent modification with 1598,132 events at the genome scale (13.8% of the total SNPs), while G/C transversions occurred 616,675 times (5.3% of the total SNPs). A/C and G/T transversions were present at intermediate and very similar frequencies with 1,118,093 and 1,118,478 events at the genome scale (9.65% of the total SNPs, respectively). In order to validate these data, the SNPs identified in this study where compared to the SolCAP Infinium High Confidence SNPs available at the PGSC website (http://solanaceae.plantbiology.msu.edu/pgsc_download.shtml). These data were produced by RNA-sequencing of Atlantic, Premier Russet, and Snowden potato cultivars. Our results corroborated 40501 of the 70326 SNPs present in the SolCAP database (Table 2). At the chromosome scale, the percentage of retrieved SNPs ranged from 45 to 72% for chromosome 2 and 6, respectively. Variants intrinsic to the Desiree genome were evaluated by filtering out the polymorphisms between cv. Desiree and the DM reference (Desiree vs. DM) that were homozygous in cv. Desiree (Desiree vs. Desiree) (Table 3). 7,768,568 SNPs and 713,986 indels were identified at the scale of the genome. The proportions of retrieved polymorphisms compared to the global analysis were 70% and 67% for SNPs and indels, respectively, at the scale of the genome thus confirming the strong intrinsic heterozygosity of the cultivar (Table 3). At the chromosomic scale, the proportions of retrieved SNPs ranged from 51% to 81% on chromosome 5 and 3, respectively, while retrieved indels ranged from 57% to 74% on the same chromosomes.

**Table 2:** Confirmed SNPs in comparison with previously identified SNPs in 3 different cultivars (Atlantic, Premier Russet and Snowden).

	SolCAP SNPs	Confirmed SNPs	Percentage
Ch00	777	276	35.52
Ch01	8562	5279	61.66
Ch02	8217	3708	45.13
Ch03	7092	4294	60.55
Ch04	6168	3168	51.36
Ch05	5706	2868	50.26
Ch06	5178	3764	72.69
Ch07	5610	2988	53.26
Ch08	4829	2850	59.02
Ch09	4905	3118	63.57
Ch10	4001	2875	71.86
Ch11	4615	2806	60.80
Ch12	4666	2507	53.73
Total	70,326	40,501	57.59

**Table 3:** Analysis of polymorphisms intrinsic to *Solanum tuberosum* cv. Desiree.

	Desiree vs. DM + Desiree vs. Desiree		Desiree vs. Desiree		% of retrieved polymorphisms	
	SNPs	Indels	SNPs	Indels	SNPs	Indels
Ch00	403,649	30,646	241,562	13,993	60	46
Ch01	1,439,553	131,669	959,934	88,741	67	67
Ch02	702,731	78,552	461,731	54,650	66	70
Ch03	758,724	88,096	614,303	65,406	81	74
Ch04	1,024,125	96,289	672,615	60,591	66	63
Ch05	863,455	78,184	444,273	44,901	51	57
Ch06	941,865	95,635	665,626	66,587	71	70
Ch07	827,253	80,630	657,147	57,404	79	71
Ch08	884,154	82,726	598,200	54,822	68	66
Ch09	957,817	84,646	728,418	60,030	76	71
Ch10	612,064	63,440	398,627	39,301	65	62
Ch11	765,122	73,019	537,495	50,106	70	69
Ch12	986,050	82,875	788,637	57,454	80	69
Total	11,166,562	1,066,407	7,768,568	713,986	70	67

Variations including both divergences between the reference genome (Desiree vs. DM) and the variability intrinsic to the Desiree genome (Desiree vs. Desiree) were compared with the intrinsic variability alone (Desiree vs. Desiree). The percentages of retrieved variations in Desiree vs. Desiree at the genome scale and for each chromosome are indicated.

**Figure 1:**
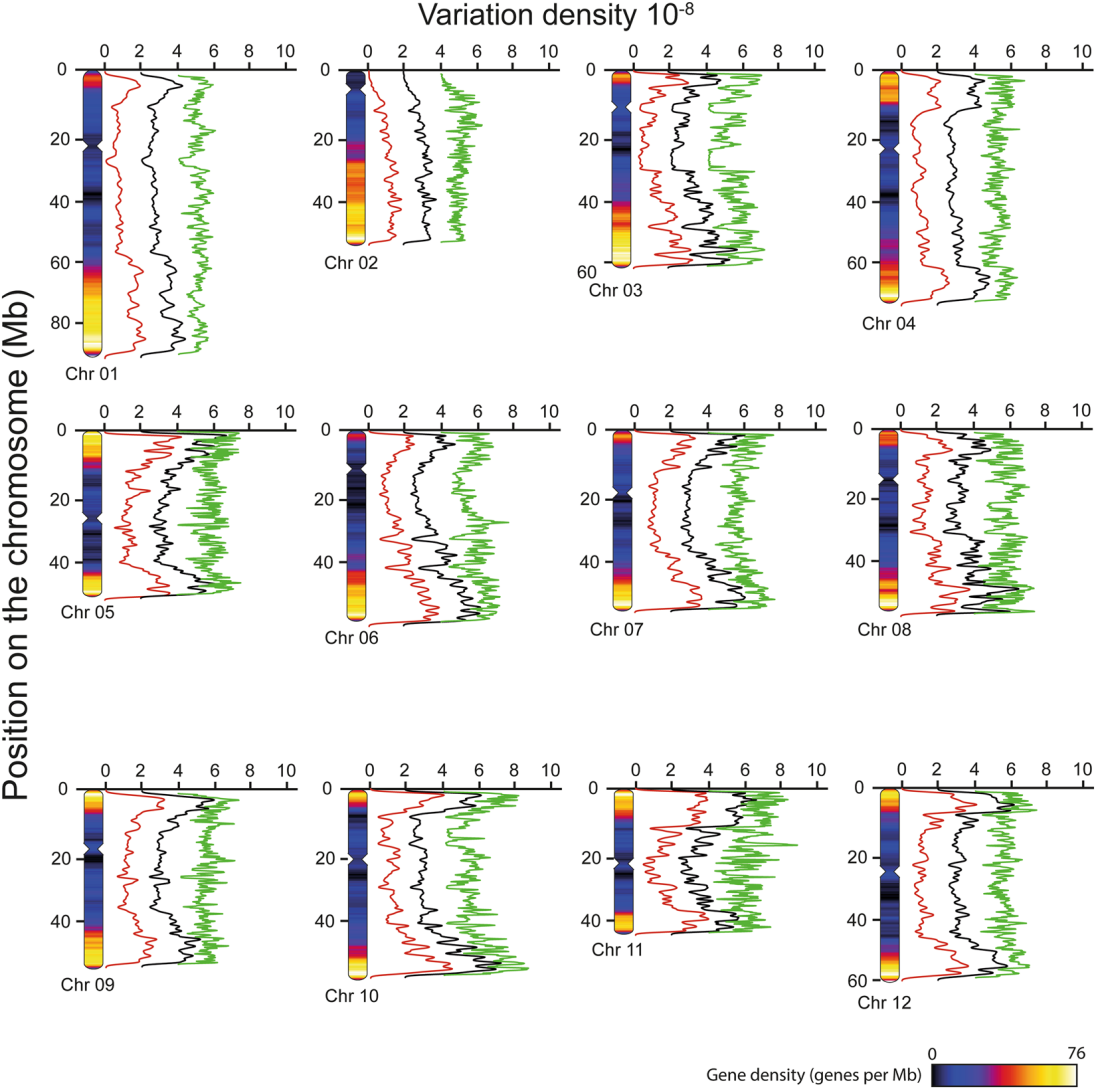
Physical polymorphism map of the potato cv. Desiree genome. Ideograms of the 12 pseudochromosomes of potato *cv* Desiree were schemed based on the released DM reference genome (3). Heat maps of gene density (genes.Mb^−1^) were constructed with the use of the “lattice” (densityplot function) and “Sushi” (plotbed function) R packages and are displayed within each chromosome. SNPs, insertions and deletions were identified by variant calling on sequence alignment of the resequenced Desiree cultivar and the DM reference genome. Polymorphism density was estimated with the use of the “lattice” (densityplot function) and “Sushi” (plotbed function) R packages and plotted next to each chromosome. The x-axis corresponds to SNP (green line), insertion (black line) and deletion (red line) densities displayed in number of variations.bp^−1^. Insertion and deletion densities were plotted with x-offsets of 2.10^−8^ and 4.10^−8^, respectively. The y-axis represents the length of each chromosome in Mb.

To investigate the local distribution of polymorphisms, a physical map of the 12 potato chromosomes was constructed based on the DM reference genome (Fig. 1). Variant calling data were processed with the use of the “lattice” and “Sushi” R packages to illustrate polymorphism and gene densities (Fig. 1). As expected, gene density was greater at chromosome edges when compared to centromere regions (Fig. 1)^3^. Although not correlated at the scale of individual chromosomes, SNP and indel densities followed similar local distribution patterns (Fig. 1). Furthermore, polymorphism density was very low in some regions including the central part of chromosome 7 and 12 as well as in the regions ranging from 10 to 60 Mb and 15 to 30 Mb of chromosome 1 and 3, respectively (Fig. 1). On the other hand, SNP enrichment could be observed in some regions, especially at the edges of chromosome 7 and 10 (Fig. 1). Moreover, the global density of variations was correlated with gene density, particularly in chromosome 1, 4, and 12.

**Figure 2:**
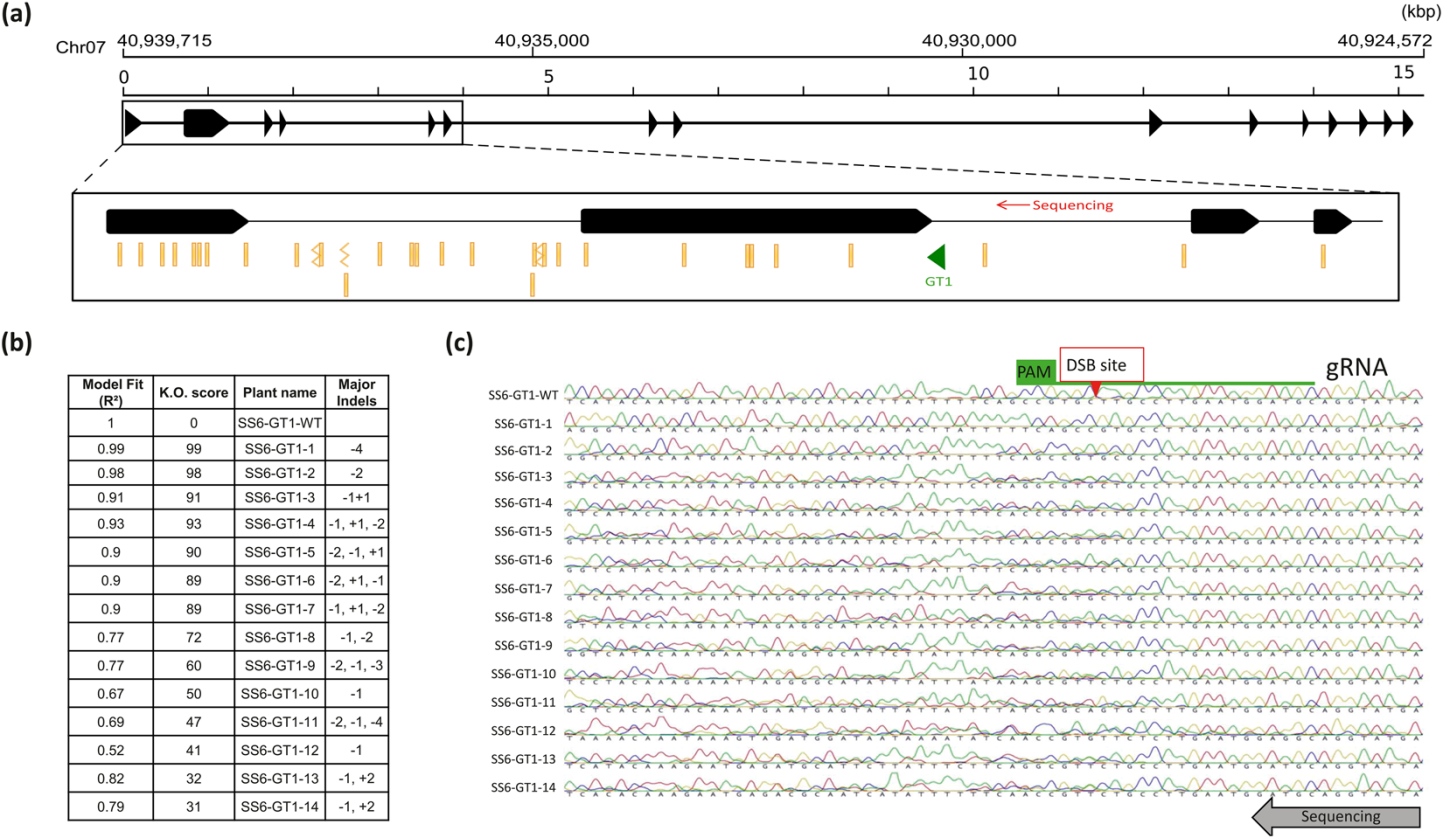
Targeted knock-out mutagenesis of the *SS6* gene in the tetraploid potato, cv. Desiree. (**a**) Schematic representation of the SS6 CDS organization. Gene coordinates on chromosome 7 are indicated at the top of the figure. The inset shows an enlargement of the four first *SS6* exons and the position of the targeted sequence GT1 (green arrow). Sequence polymorphisms are indicated by orange signs. Straight line ticks correspond to substitutions or deletions. Broken line ticks correspond to insertions. Sanger sequencing of the PCR amplification products of *SS6* from transformed plants was performed unidirectionally. The location of the primer is indicated by a red arrow. (**b**) List of the genetically modified plants and the corresponding model fitting, K.O. (knock-out) mutations scores and predicted major indels obtained from the ICE tool analysis. (**c**) Sequence analysis of the targeted *SS6* region in the wild-type (SS6-GT1-WT) and 14 genetically transformed plants (SS6-GT-1 to -14). The electrophoregrams were obtained by Sanger sequencing. The sequences from SS6-GT1-2 to SS6-GT1-14 display superimposed electrophoregram signals caused by the heterogeneity of the indel mutations among the four gene copies. On the other hand, SS6-GT1-WT harbors a unique electrophoregram indicating the homozygosity of the wild type allele and SS6-GT1-1 shows homozygosity for a 4 bp deletion at the predicted double-strand break (DSB) site. The gRNA is depicted in green. PAM: protospacer adjacent motif.

In order to illustrate the efficiency of knowing which DNA regions should be avoided when designing gRNAs for tetra-allelic gene knockout in tetraploid potato, we used the data reported here to mutate the *SS6* gene in *S*. *tuberosum*, cv. Desiree (Fig. 2). A target region (GT1), displaying good predicted efficiency and specificity for CRISPR/Cas9 gene inactivation, was identified in a polymorphism free sequence at the end of the second exon of the *SS6* gene (Fig. 2a). The corresponding gRNA was cloned into pDIRECT_22a by golden gate reaction^18^. The latter construct was used for Agrobacterium-mediated transformation of potato explants and fourteen regenerated plant transformants were selected by PCR amplification of the Cas9 gene present on pDIRECT_22a. A second PCR amplification of the targeted region of *SS6* was followed by Sanger sequencing and electrophoregram fitting analysis with the use of the ICE tool (https://ice.synthego.com/) (Fig. 2b,c). Among the fourteen transformed plants, seven (SS6-GT1-1 to SS6-GT1-7) showed a coefficient of determination (*R*^2^) above 0.9 accompanied by KO (knock-out) -scores ranging from 89 to 99 (Fig. 2b). The seven other transformants (SS6-GT1-8 to SS6-GT1-14) displayed a *R*^2^ ranging from 0.52 to 0.82 and KO-scores between 31 and 72 (Fig. 2b). While the probability that lines SS6-GT1-1 to SS6-GT1-7 are knock out mutants is very high, only SS6-GT1-1 harbored the same mutation on each of the four *SS6* alleles resulting in a clear non-superimposed electrophoregram with a 4bp deletion at the site of predicted DSB (Fig. 2b,c). On the other hand, lines SS6-GT1-2 to -7 carried a mix of predicted indels of one or two bp in length (Fig. 2b).

## Discussion

Regardless of the genome editing strategy, targeting a polymorphism-free DNA region is crucial for generating tetra-allelic mutations in heterozygous polyploids. In functional genomic approaches, target design is commonly performed based on the released genome sequence of the organism. The available sequence of the potato genome was obtained from a homozygous doubled-monoploid (DM) and a heterozygous diploid (RH) clone. In the present study, we resequenced that of the laboratory cultivar Desiree which was historically chosen for its ease of transformation and is still widely used for biomolecular studies of the potato. Variant calling analysis showed occurrences of 1 SNP/68 bp, 1 insertion/1173 bp and 1 deletion/1753 bp. These variations comprised both sequence divergences from the reference genome (Desiree vs. DM) and the variability intrinsic to the Desiree genome (Desiree vs. Desiree). Sequence variations between Desiree and DM genomes have to be taken into account for determining DNA binding sequences in gene editing approaches. However, when these polymorphisms are homozygous within the studied potato line, sequences overlapping the corresponding positions can be designed as targets for homozygous gene editing. Thus, we filtered out the polymorphisms between cv. Desiree and the DM reference (Desiree vs. DM) that were homozygous in cv. Desiree (Desiree vs. Desiree). 70% and 67% for SNPs and indels, respectively, were retrieved in this analysis indicating that particular attention must be paid during target design for multiallelic gene editing of potato. For instance, inducing DSBs with the use of CRISPR/Cas9 requires designing a ≈20bp guide sequence (gRNA) to pair with target DNA. The presence of SNPs or Indels within the targeted sequence alters the binding of gRNAs thus misbalancing the on-/off-target Cas9 activity. Individual gene sequencing can be performed to overcome this issue. However, this approach is time consuming and not compatible with high throughput functional genomics.

Here, we used these data to select a target region free of variants for inactivating the *SS6* gene by CRISPR/Cas9. Among fourteen transformed plants, seven showed a very high probability of knock-out mutation on each of the four *SS6* alleles. Moreover, one mutant line contained identical deletions on each of the four chromosomes. Further investigation of the starch structure and morphology from these mutant lines will reveal the function of SS6 that remains to be deciphered. Furthermore, the inactivation of this enzyme may lead to modifications of starch properties potentially resulting in industrial applications. The data reported here will allow improving the design workflows for gene editing of the routinely used cultivar Desiree. Potato is the fourth most important staple crop in the world implying important breeding efforts in a climate changing context concomitant with increasing global population. Thus, accelerating research on potato genetics and physiology will likely lead to key fundamental discoveries as well as important industrial applications.

## Methods

### Plant material and growth condition

Explants of *Solanum tuberosum* cultivar Desiree were provided by the biological resource center BrACySol (IGEPP INRA). Plants were cultivated on Murashige and skoog medium^19^ including vitamins and 8 g.l^−1^ Plant Agar (Duchefa^®^) in a growth chamber with a 16h-light (80 µmol.m^−2^.s^−1^)/8h-dark photoperiod, at 20 °C during 3 weeks. Plants were propagated *in vitro* every 3 weeks by transplantation of the terminal bud.

### Sample preparation and resequencing

Leaf DNA from tetraploïd *Solanum tuberosum* L. cv. Desiree was extracted with the use of the Nucleospin plant II kit (Macherey Nagel). A shotgun library was constructed from 100 ng of DNA using “Truseq DNA nano sample prep” (Illumina Inc.). Validation was then realized using the “DNA HS Assay kit” (Agilent). Mean size of fragments was 550 bp. The library was then sequenced using an Illumina HiSeq 2500 generating 99,713,534 paired-end reads of 2 × 150bp corresponding to a 35X mean coverage of the 844 Mb potato genome. Read quality have been analyzed using FastQC v0.11.5, giving 90.44% of reads with a Phred Q quality score >30.

### Read alignment and variant calling

The following steps were performed using the Geneious 11.1.2 software (https://www.geneious.com)^20^. Poor quality data were trimmed from the ends of the paired reads with an error probability limit of 0.05. Paired reads were then mapped to the reference genome pseudomolecules using bowtie2 mapper^21^ set to high sensitivity resulting in the assembly of 167,343,472 of the 199,427,068 reads on 13 contigs. Variant calling was performed on the whole assembled contigs using the Geneious variant finder tool with the following settings: variations positions must be covered by at least 8 reads with a frequency above 0.05. The variant *P*-value and the strand-bias *P*-value limits were set to 10^−5^. This first analysis combined variations within the genome of the potato cultivar Desiree (Desiree vs. Desiree) and variations between the DM reference genome and that of cv. Desiree (Desiree vs. DM). A second analysis was performed as described above except that the divergences from the DM reference genome (Desiree vs. DM) were filtered out by excluding the reference sequence in the variant calling analysis.

### Target design and plasmid construction

The full CDS of *StSS6*, located on chromosome 7 (position 40,939,715 to 40,924,572, PGSC_DM_v4.03_pseudomolecules, http://solanaceae.plantbiology.msu.edu/) was reconstructed using the CDS of the *SS6* gene from *Solanum lycopersicum* (accession number: Solyc07g042830.2, http://solgenomics.net) as a template. Both genomic regions were aligned in Geneious 11.1.2 and the partially annotated CDS from *S*. *tuberosum* (position 40,939,715 to 40,932,630, accession number: PGSC0003DMG402013540, http://solanaceae.plantbiology.msu.edu/) described in^22^ was extended according to the corresponding gene structure of *S*. *lycopersicum*. Intron splicing signals were positioned with respect to the tomato gene and gene structure was confirmed by in silico translation of the complete CDS. The obtained protein sequence displayed 100% identity with the full length annotated SS6 protein sequence (XP_006353746.1, https://www.ncbi.nlm.nih.gov/protein/).

The targeted sequence for the inactivation of *SS6* (GT1: 5′-GCATCCATTCAAGGCAAGCA-3′) was defined to meet with a high predicted efficiency (activity score of 0.69) and specificity (score of 100%) with the use of the geneious 11.1.2 software and a frame shift probability (microscore of 67.15) using the CRISPR-P toolbox (http://crispr.hzau.edu.cn/CRISPR2/). To favor targeting of the four *SS6* alleles, the absence of polymorphisms in the targeted sequence was confirmed using the variant calling data generated in this study. The gRNA was cloned into the pDIRECT_22a plasmid by golden gate reaction using the AtU6 sense gRNA (5′-GATTGCATCCATTCAAGGCAAGCA) and antisense gRNA (5′-AAACTGCTTGCCTTGAATGGATGC) oligonucleotides purchased from Eurogentec (Belgium) and according to protocol 2B from^18^. The resulting vector pDIRECT_22a:GT1 contained a kanamycin resistance gene, a plant codon-optimized Cas9 gene and the sgRNA targeting the GT1 sequence of *SS6*.

### Agrobacterium-mediated transformation

*Agrobacterium tumefaciens* C58pMP90 was transformed with the pDIRECT-22a:GT1 plasmid and cultivated during 48 h at 28 °C in LB medium containing 50 mg/L rifampicin, 25 mg/L gentamicin and 50 mg/L spectinomycin. Potato (cv. Desiree) plants were cultivated on solid Murashige and Skoog (MS) medium supplemented with vitamins (Duchefa, The Netherlands) and containing 2% sucrose (medium A) with a photoperiod of 16-h light (65 µmol photons.m^2^.sec^−1^)/8-h dark at 20 °C. Stem and petiole explants were cut from 4-week-old plants prior to incubation on sterile MS plates including vitamins (Duchefa, The Netherlands), 2% sucrose, 1 mg/L indole-3-acetic acid (Sigma-Aldrich, USA), 1 mg/L zeatin-riboside (Sigma-Aldrich, USA) and 1 mg/L gibberellin A3 (Sigma-Aldrich, USA) (medium B) during 24h in the same conditions of light and temperature. Explants were then dipped in a suspension of *A*. *tumefaciens* containing pDIRECT-22a-GT1 (OD_600_ = 0.2) prior to incubation on medium B at 25 °C in the dark during 48 h. Explants were washed with sterile water and 1X MS medium containing 2% sucrose, 500 mg/L cefotaxime, 500 mg/L Ticarcillin/clavulanic acid and 50 mg/L kanamycin prior to be placed on solid medium B during 2 weeks under the aforementioned conditions until the apparition of calli. Explants were then subcultured every two weeks on medium C (same as medium B with a 10-fold decreased concentration of Indolo-3-acetic acid) until shoot regeneration.

### Plant selection and genotyping

Regenerated shoots were isolated and cultivated on solid medium A. Mature leaves from 14 transformed plants were cut and genomic DNA was isolated using the cetyltrimethylammonium bromide (CTAB) method^23^. In order to confirm the integration of the plasmid, the Cas9 gene was amplified by PCR using the following primers: Cas9For: 5-TTCGGGCGTTCCAGTGATAC-3, Cas9-rev: 5-TATGGTGTGCAACGGTCTCC-3 and the DreamTaq DNA polymerase (Thermo Fisher Scientific, Waltham, Massachusetts). The following parameters were used for PCR reaction: denaturation for 2 min at 95 °C; 30 consecutive cycles at 95 °C for 1 min, 60 °C for 30 sec, 72 °C for 25 sec; and finally elongation at 72 °C for 10 min. The presence of mutations on the targeted sequence was investigated by PCR amplification using the following primers: GT1For: 5-TTCGGGCGTTCCAGTGATAC-3, GT1Rev: 5-TATGGTGTGCAACGGTCTCC-3 and the high fidelity DNA polymerase (kappa hifi, Roche, Basle, Switzerland) with the following parameters: denaturation for 2 min at 95 °C; 30 consecutive cycles at 95 °C for 1 min, 60 °C for 30 sec, 72 °C for 25 sec. The PCR products were sequenced by the Sanger method using GT1Rev: 5-TATGGTGTGCAACGGTCTCC-3 (Eurofins genomics, Ebersberg, Germany). Sequencing data were analyzed by comparison with the corresponding electrophoregram of the wild-type sequence using the ICE tool available at https://ice.synthego.com/.
